# Process Parameter Optimization and Straightness Error Prediction in FDM Based on PB-CCF Design

**DOI:** 10.3390/polym18161954

**Published:** 2026-08-10

**Authors:** Li Yang, Tianlu Wei, Pei Li, Jing Zhao

**Affiliations:** School of Mechanical and Vehicle Engineering, Bengbu University, Bengbu 233000, China; lipei1986yeah@bengbu.edu.cn (P.L.); zhaojingzaozao@163.com (J.Z.)

**Keywords:** fused deposition modeling, Plackett-Burman design, CCF, straightness error, response surface methodology, decision tree-genetic algorithm

## Abstract

Fused deposition modeling (FDM) involves numerous process parameters, making it difficult to efficiently identify key influencing factors and achieve accurate prediction of straightness errors in printed parts. To address these issues, this paper proposes a two-stage experimental optimization and prediction method combining Plackett–Burman (PB) design and central composite design (CCD). First, PB experiments were conducted to screen nine process parameters and identify two significant factors affecting straightness error: layer height and line width. Subsequently, a face-centered central composite design (CCF) was employed to establish a quadratic response surface regression model for analyzing the main effects and interaction effects of the selected factors. Based on 18 sets of CCF experimental data, response surface methodology (RSM) and decision tree–genetic algorithm (DT + GA) prediction models were constructed, and their generalization capabilities were evaluated using five independent reserved experiments. The results show that the quadratic regression model exhibits a coefficient of variation of 2.21% and a signal-to-noise ratio of 45.45, indicating good fitting accuracy. On the test set, RSM achieves a mean squared error (MSE) of 7.29 × 10^−6^ mm^2^ and a mean absolute error (MAE) of 0.002142 mm, both superior to the DT + GA model (MSE = 3.41 × 10^−5^ mm^2^, MAE = 0.004578 mm), representing reductions of 78.6% in MSE and 53.2% in MAE. This validates the effectiveness and superiority of the CCF-based response surface method for straightness error prediction under small-sample conditions. The proposed strategy provides a quantitative reference for the control of shape accuracy and process optimization in FDM-printed parts.

## 1. Introduction

Fused deposition modeling (FDM) is one of the most widely used additive manufacturing technologies, which manufactures three-dimensional objects by extruding thermoplastic materials layer by layer [1,2,3]. Polylactic acid (PLA), owing to its good biodegradability and low warpage tendency, has become one of the most commonly used filaments in FDM [4,5,6].

When FDM-printed parts serve as structural components, their geometric accuracy directly determines assembly quality and service performance. Among various geometric tolerances, straightness error is particularly critical; for components such as guide rails and sliders, excessive deviation can lead to motion offset, uneven fit clearance, and increased vibration [7,8]. Therefore, controlling straightness error through process parameter optimization is a key issue in FDM precision research.

Recent studies on FDM process parameters and part accuracy have been conducted from multiple perspectives, including surface quality, dimensional accuracy, mechanical properties, and shape accuracy [9]. Regarding surface quality, layer height is a decisive factor affecting roughness [10]; the coupled effects of infill pattern, layer height, and printing temperature have been systematically investigated [11,12]; post-processing techniques have also been employed for surface improvement [13]. For dimensional accuracy, a Box–Behnken design has been used to optimize layer height, printing speed, and temperature for improved part precision [14]. In terms of mechanical properties, central composite design (CCD) experiments have studied the effects of layer thickness, printing speed, and infill density [15]; infill pattern and ratio have been found to significantly affect part strength [16,17]. For shape accuracy, layer height, infill ratio, and printing temperature have been identified as core variables affecting warpage and flatness [18,19,20]. The straightness of digital light processing (DLP)-printed parts has been evaluated, and results indicate that resin color significantly influences this geometric tolerance [21]. Meanwhile, machine learning methods have also been introduced for FDM accuracy prediction in recent years: artificial neural networks (ANN) have been employed to predict the dimensional accuracy of PLA parts [22]; adaptive neuro-fuzzy inference system (ANFIS) has been established for predictive modeling of PLA printing performance in FDM [23]; a whale algorithm-optimized support vector regression model combined with a multi-objective optimization framework has been proposed for balanced optimization of dimensional accuracy and printing efficiency in FDM [24]. However, the aforementioned studies have primarily focused on conventional metrics such as surface quality, dimensional accuracy, or mechanical properties, while systematic investigation of straightness error through FDM process parameters remains limited.

Despite these advances, several critical issues remain. FDM platforms have more than ten adjustable parameters, yet most studies empirically preselect only 3–5 for optimization, lacking an efficient method to identify key factors from numerous candidates. High-quality experimental data are often limited due to cost and time constraints; data-driven models risk overfitting under small-sample conditions, while the prediction accuracy of response surface methods in small-sample scenarios also lacks systematic validation. Furthermore, research on predicting and controlling straightness error through process parameters remains insufficient.

To address these issues, this paper proposes a three-stage strategy of “Plackett–Burman (PB) screening—face-centered central composite design (CCF) response surface modeling—response surface methodology (RSM)/decision tree–genetic algorithm (DT + GA) prediction comparison”: PB design is employed to screen significant factors from nine parameters; face-centered CCF is used to establish a quadratic response surface model; and RSM [25,26,27] and DT + GA prediction models [28] are constructed and compared using 18 CCF data sets. Compared with previous studies, the novelty of this work lies in three aspects: (1) systematically investigating the parametric optimization of straightness error in FDM-printed parts, distinguishing from RSM-based optimization studies focusing on surface quality [10,11,12,13] or mechanical properties [15,16,17], as well as works that developed novel extrusion systems [29]; (2) employing a PB + CCF two-stage strategy for systematic parameter screening and response surface modeling; and (3) providing a quantitative comparison between RSM and DT + GA under small-sample conditions to guide model selection in data-limited engineering scenarios.

## 2. Materials and Methods

### 2.1. Materials

The material used in the experiments was PLA Matte filament (Shenzhen Tuozhu Technology Co., Ltd., Shenzhen, China), with a filament diameter of 1.75 mm, density of 1.31 g/cm^3^, Vicat softening temperature of 63 °C, and melting temperature of 163 °C.

### 2.2. Equipment and Instruments

**Figure 1:**
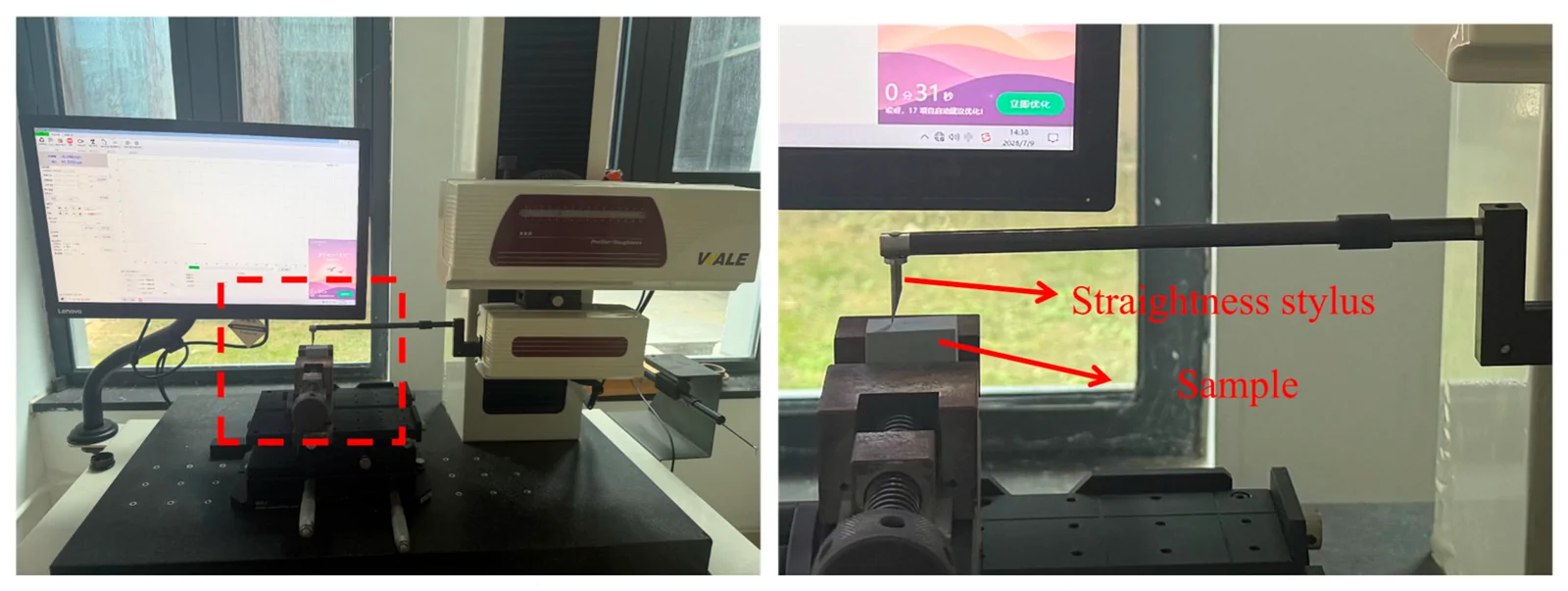
Profilometer. The red box indicates the measured region corresponding to the enlarged detail shown on the right.

A Bambu Lab P1S 3D printer (Shenzhen Tuozhu Technology Co., Ltd., Shenzhen, China) equipped with a standard 0.4 mm nozzle was used for specimen fabrication. The straightness error of the printed parts was measured using a 2000 series roughness profiler (Shaanxi Will Precision Mechanical and Electrical Technology Co., Ltd., Xi’an, China), which has a vertical resolution of 0.0001 mm, as shown in Figure 1.

### 2.3. Printing Model and Characterization

**Figure 2:**
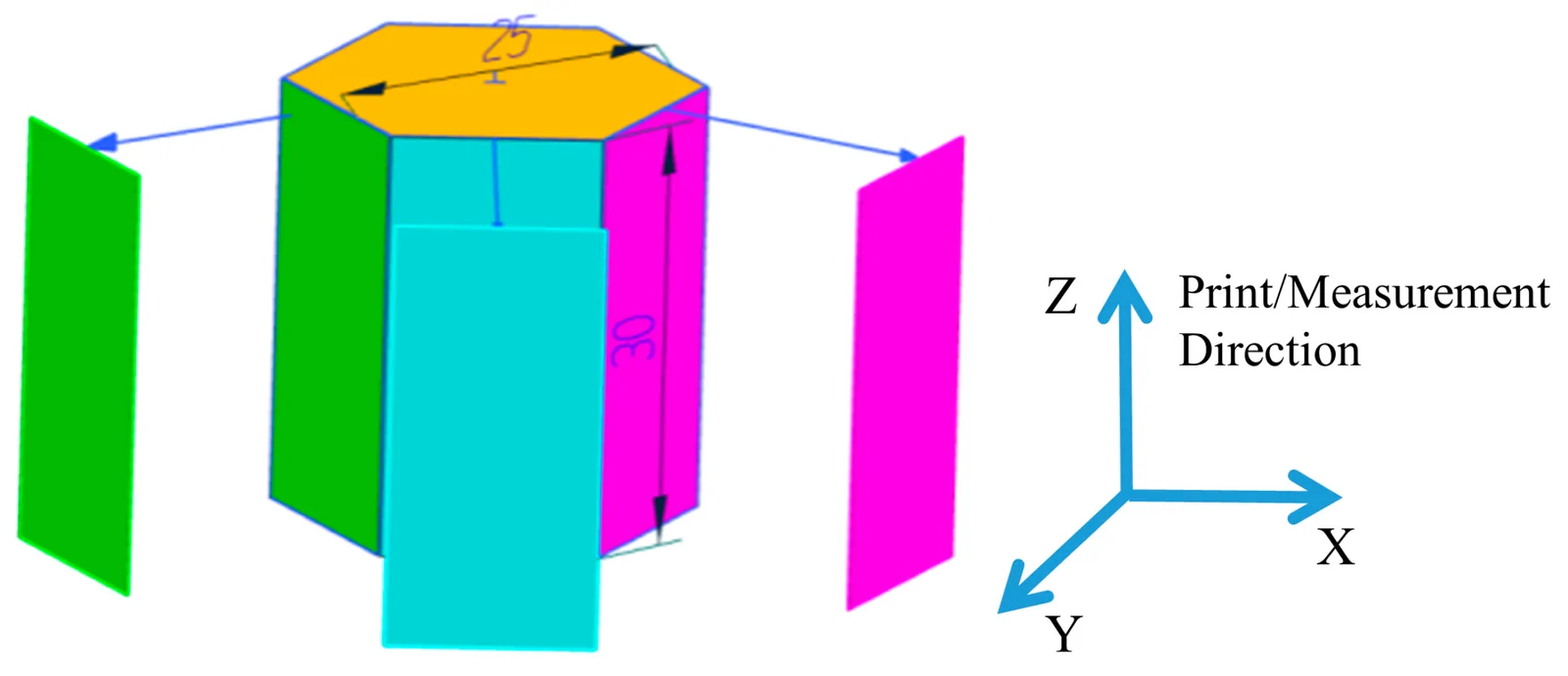
Printing model and measurement surfaces. The blue, green, and purple faces represent the front, left, and right measurement surfaces, respectively.

The printed model was designed as a hexagonal prism with a distance across flats of 25 mm and a height of 30 mm. The dimensions and three measurement surfaces are shown in Figure 2. The printing direction and measurement direction are consistent, both along the *Z*-axis of the model.

To ensure experimental consistency, all specimens were printed using the same spool of material to avoid batch-to-batch variations. To eliminate potential thermal accumulation or cross-interference caused by the nozzle moving between different parts, all specimens in this study were printed one at a time, with each model positioned at the center of the build platform. The printing sequence for all PB and CCF experimental runs was randomized according to the Design-Expert-generated run order to eliminate systematic time-related errors. After each print was completed and the print head moved away, the specimen was allowed to cool naturally in the build chamber for 40 min before removal, and the next model was then printed following the same procedure. This strategy avoided residual stress variations caused by rapid cooling. All printed specimens were then conditioned in the laboratory ambient environment for 48 h prior to measurement to stabilize internal stresses and dimensions.

For the PB experimental specimens, three prismatic surfaces—the front, left, and right faces—were selected to evaluate the straightness errors from different orientations. Measurements were conducted along the height direction of the model. On each face, three measurement lines were equally spaced along the height direction, and each line was measured once. Prior to measurement, the profilometer was calibrated using standard gauge blocks and ceramic standard spheres according to the manufacturer’s standard operating procedure. The straightness error was evaluated in accordance with ISO 12780-1 [30]: the dedicated software denoised the raw profile data using a Gaussian filter, automatically fitted a reference line, and calculated the sum of the absolute values of the maximum positive and negative deviations from this reference line as the straightness error. The arithmetic mean of the three measured lines was taken as the final straightness error for that face. The sampling length was set to 20 mm with a sampling interval of 0.2 μm.

### 2.4. Experimental Design

Design-Expert 12 (Stat-Ease, Inc., Minneapolis, MN, USA) was used to generate the Plackett–Burman design matrix and the CCF response surface model, and to perform ANOVA and regression fitting on the experimental results. The first stage was the Plackett–Burman (PB) screening experiment. Based on literature reviews [1,2] and FDM process mechanisms, nine candidate process parameters were selected from four dimensions: thermal field, extrusion process, geometric configuration, and motion status. Nozzle temperature (H) and bed temperature (J) control the thermal boundary conditions, which have been shown to significantly influence part distortion and thermal stress [20]; extrusion flow rate coefficient (G) regulates material supply stability and directly affects filament deposition uniformity [11]; layer height (A), line width (B), wall line count (C), sparse infill density (D), and infill angle (E) collectively determine the internal structure and cross-sectional geometry of the part [10,14]; wall printing speed (F) affects the deposition dynamics and thermal history of the extruded filaments [27]. The factor levels are listed in Table 1.

The second stage employed a face-centered central composite design (hereinafter referred to as CCF) for response surface modeling and significance verification. Among the three types of CCD, CCF (α = ±1) was selected because the axial points of CCC exceed the feasible physical limits, while CCI sacrifices boundary information. In contrast, CCF ensures that all experimental points remain within the feasible range while preserving the corner information of the design space, which is more suitable for the boundary sample learning required by the subsequent decision tree (DT) model. Furthermore, to compensate for the lack of rotatability in CCF and to ensure the validity of the lack-of-fit test, 10 replicated center points were incorporated into the design. This provides sufficient degrees of freedom for estimating pure error and balances the global prediction variance by reinforcing the weighting of the central region, thereby establishing a solid model foundation for the stable optimization of the genetic algorithm (GA). The CCF factor levels are listed in Table 2.

## 3. Results

### 3.1. PB Experimental Design and Results Analysis

**Figure 3:**
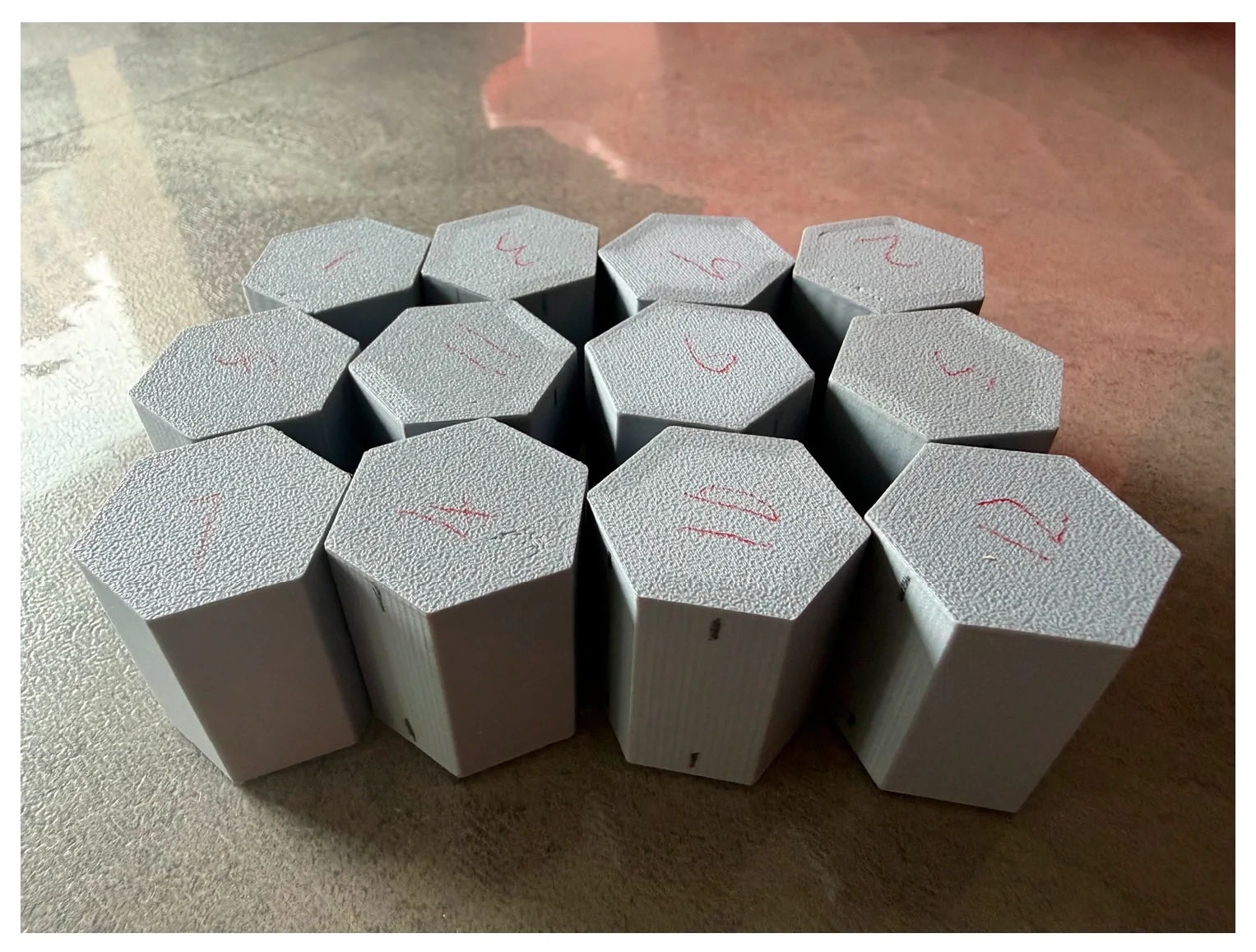
Printed models for screening experiments.

**Figure 4:**
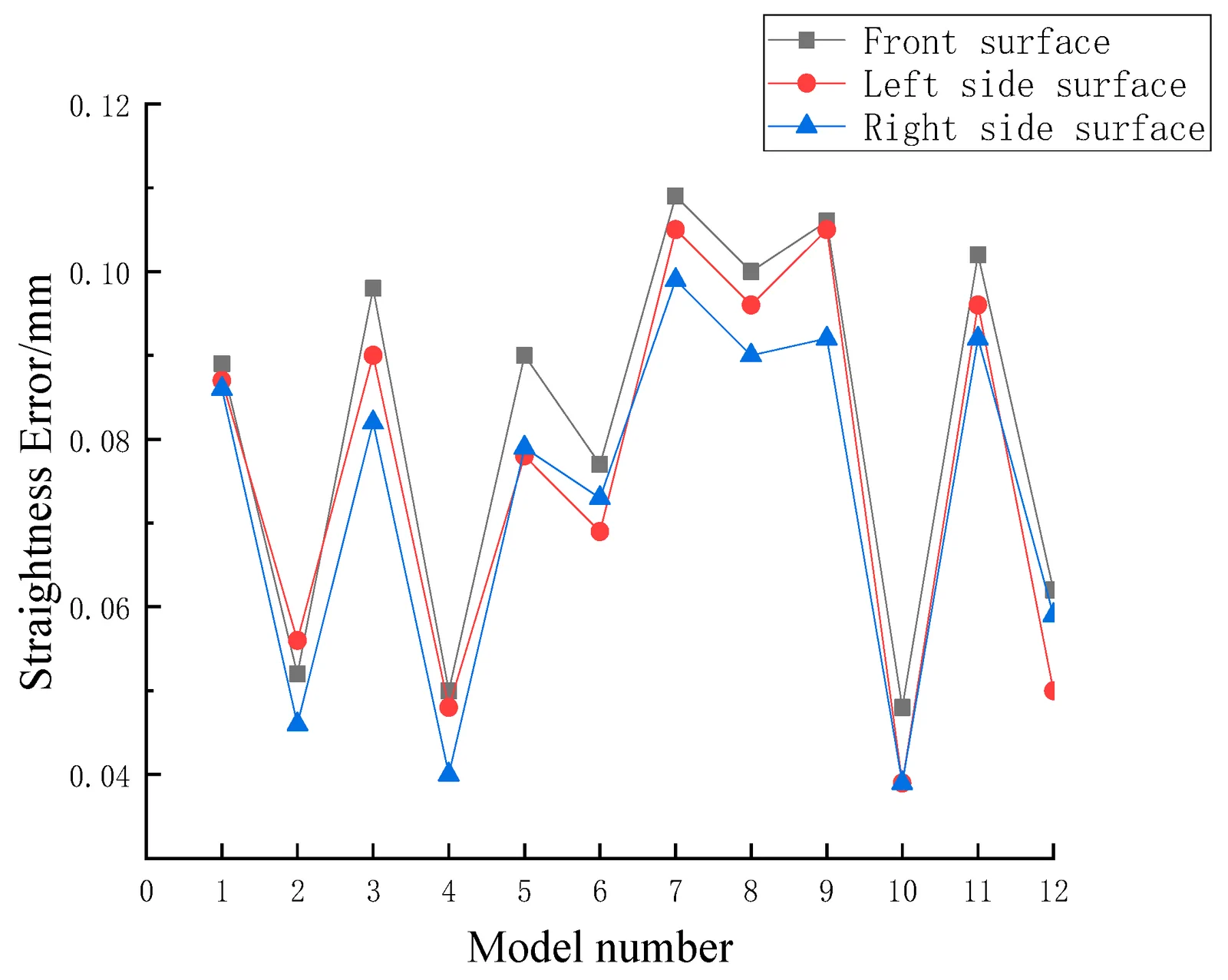
Straightness errors of the 12 PB experimental models.

A Plackett–Burman design matrix consisting of 12 experimental runs was generated using Design-Expert software 12. According to this matrix, 12 experimental models were printed for subsequent analysis, as shown in Figure 3. The measurement results are presented in Figure 4. The straightness errors of the three prismatic surfaces of each model exhibited highly consistent trends with variations in process parameters, indicating that the response patterns of different measurement surfaces were directionally consistent. Therefore, the front face data were selected as representative for subsequent significance analysis, which did not affect the screening conclusions for the key factors.

**Figure 5:**
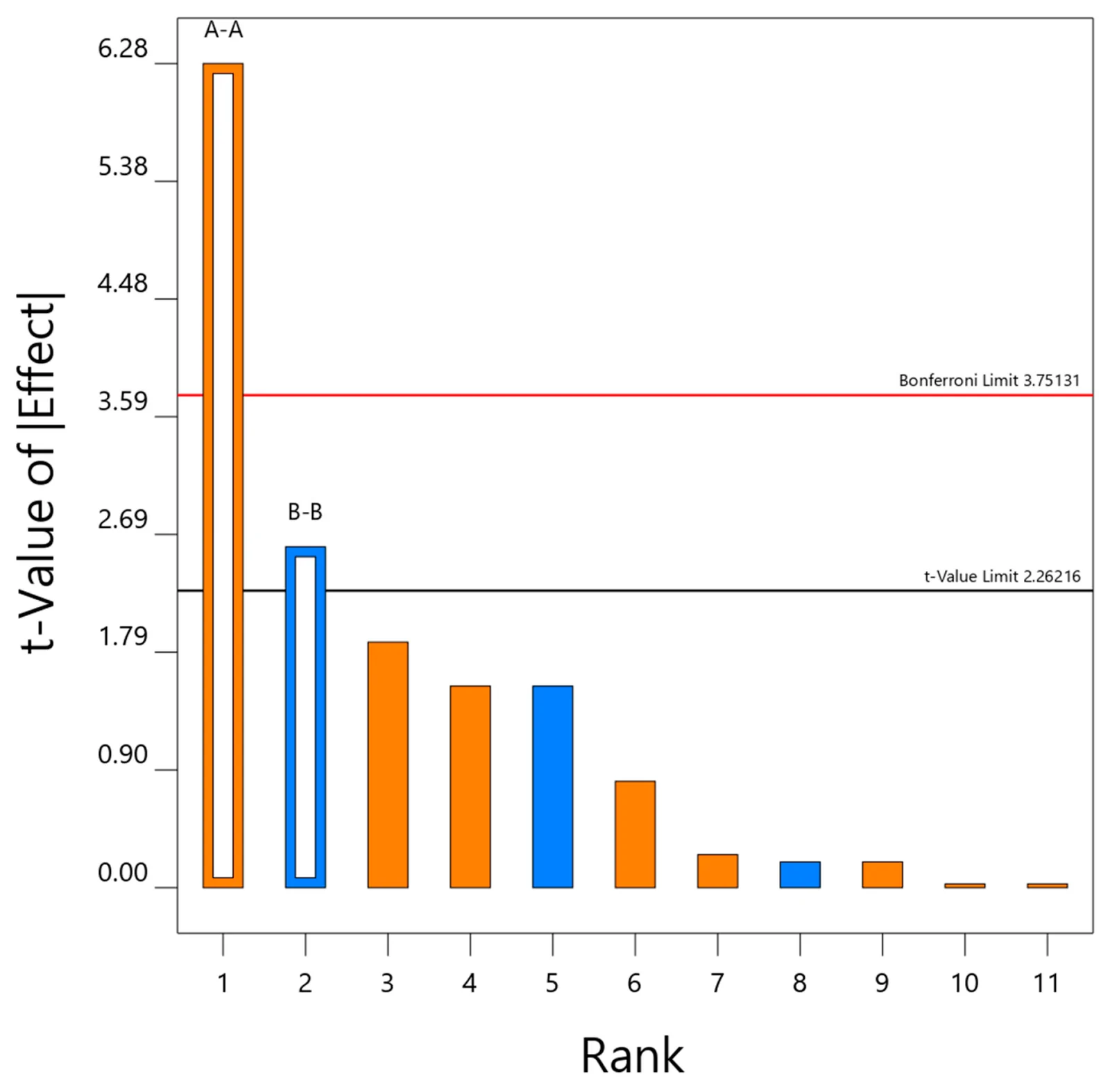
Pareto plot of factor effects in the PB experiment.

Analysis of variance (ANOVA) was performed on the PB experimental results to identify process parameters with significant effects on straightness error. Since the PB experiment was primarily intended for screening purposes with relatively low resolution, the variance model was simplified by removing non-significant terms. The Pareto plot is shown in Figure 5. The t-value of factor A (layer height) was 6.28, with a bar far above the Bonferroni limit (3.75131), indicating that it is a highly significant factor (*p* < 0.01), representing the core factor in the entire experiment with the greatest influence on the results. The t-value of factor B (line width) was 2.68, with a bar below the Bonferroni limit (3.75131) but significantly above the t-value critical line (2.26216), indicating that it is a significant factor (*p* < 0.05) that must also be retained in subsequent experiments.

The ANOVA results are summarized in Table 3.

As shown in Table 3, the F-value of the regression model for straightness error was 23.07, with a *p*-value of 0.0003 (*p* < 0.01), indicating that the model is statistically highly significant. The coefficient of determination R^2^ was 0.8368, and the adjusted R^2^ was 0.8005, indicating that the model can explain approximately 80.057% of the response variation. For a PB design intended for screening purposes, this goodness-of-fit is within an acceptable range. The adequate precision of the model reached 10.2452, far above the critical value of 4, indicating that the model possesses excellent predictive capability and can be used for subsequent response surface optimization. Among all factors examined, the *p*-values of factors A and B were both less than 0.05, confirming their significant effects on straightness error.

### 3.2. CCF Experimental Design and Results Analysis

**Figure 6:**
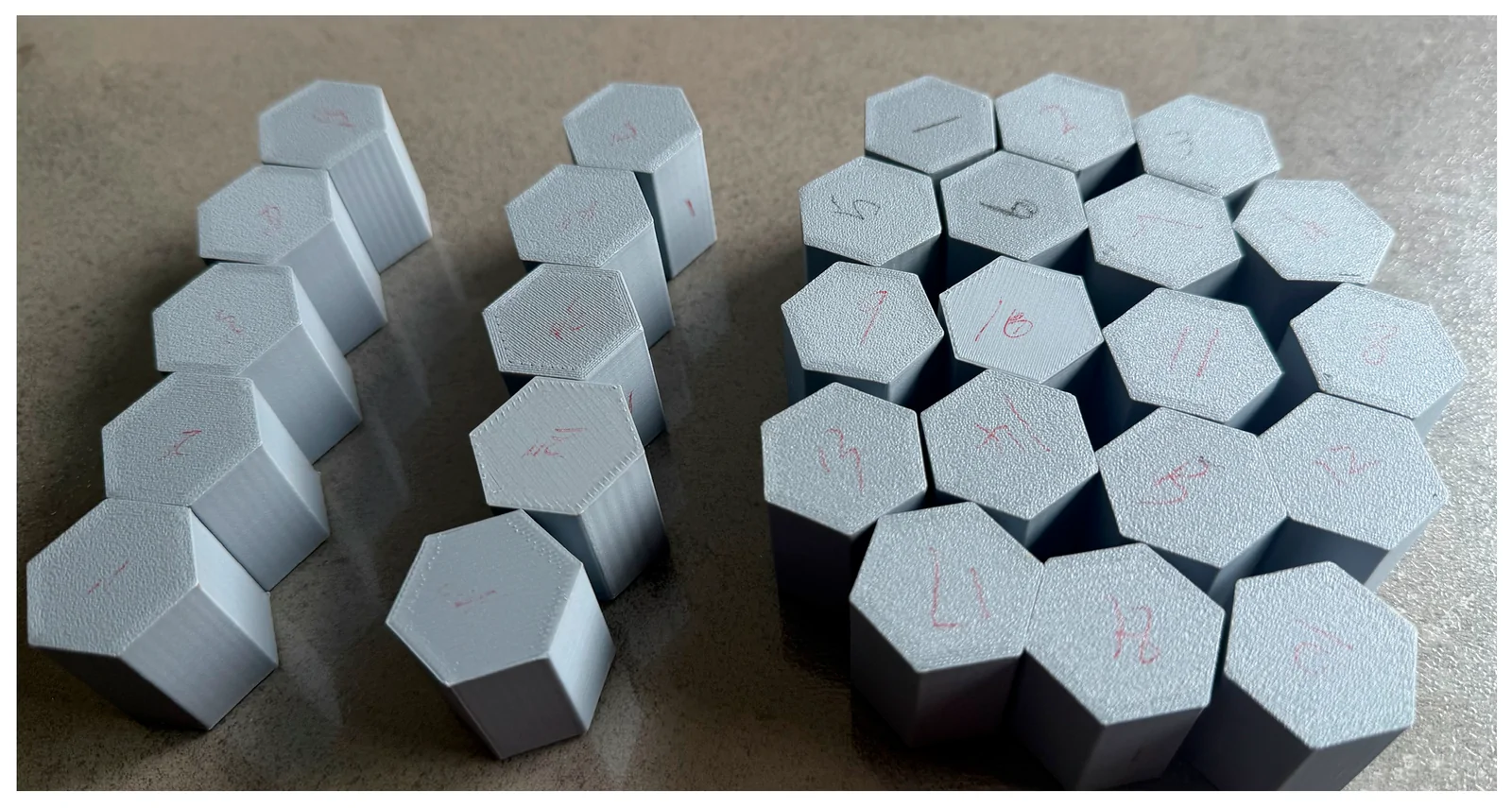
Printed specimens for CCF experimental runs (18 models), test set evaluation (5 models), and confirmation experiments under optimal conditions (5 models).

Based on the PB experimental results and the CCF experimental scheme described in Section 2.4 (factor levels shown in Table 2), a total of 18 experimental runs were completed. In addition, five test experiments with different parameter combinations were arranged (Test Point 1: layer height = 0.14 mm, line width = 0.42 mm; Test Point 2: layer height = 0.18 mm, line width = 0.48 mm; Test Point 3: layer height = 0.12 mm, line width = 0.40 mm; Test Point 4: layer height = 0.20 mm, line width = 0.50 mm; Test Point 5: layer height = 0.10 mm, line width = 0.55 mm). All experimental runs were randomized to eliminate systematic time-related errors. The experimental scheme is presented in Table 4, and the printed models are shown in Figure 6. Both the 18 CCF experimental runs and the 5 validation runs used the same hexagonal prism model as the PB experiments to ensure continuity and comparability. Additionally, five confirmation experiments were also conducted under the predicted optimal process parameters; these specimens are included in Figure 6 and discussed in detail in Section 4.2. The measurement target was the straightness error along the height direction on the front face of the model.

The experimental data were imported into Design-Expert software for model fitting analysis. The fitting performances of candidate models—linear, two-factor interaction (2FI), quadratic, and cubic—were first compared. As shown in Table 5, the cubic model was non-significant and was therefore discarded. Among the statistically significant models, the quadratic model exhibited the highest adjusted R^2^ (R^2^adj) and predicted R^2^ (R^2^pred), both superior to those of the linear and 2FI models, indicating that the inclusion of interaction and quadratic terms effectively improved the model’s explanatory power. The quadratic model also achieved the highest predicted R^2^ (0.9198), demonstrating the best predictive capability for new data. Moreover, the close agreement between R^2^adj and R^2^pred (difference < 0.2) indicated good model robustness. Therefore, the quadratic model was selected for subsequent ANOVA and optimization.

**Figure 7:**
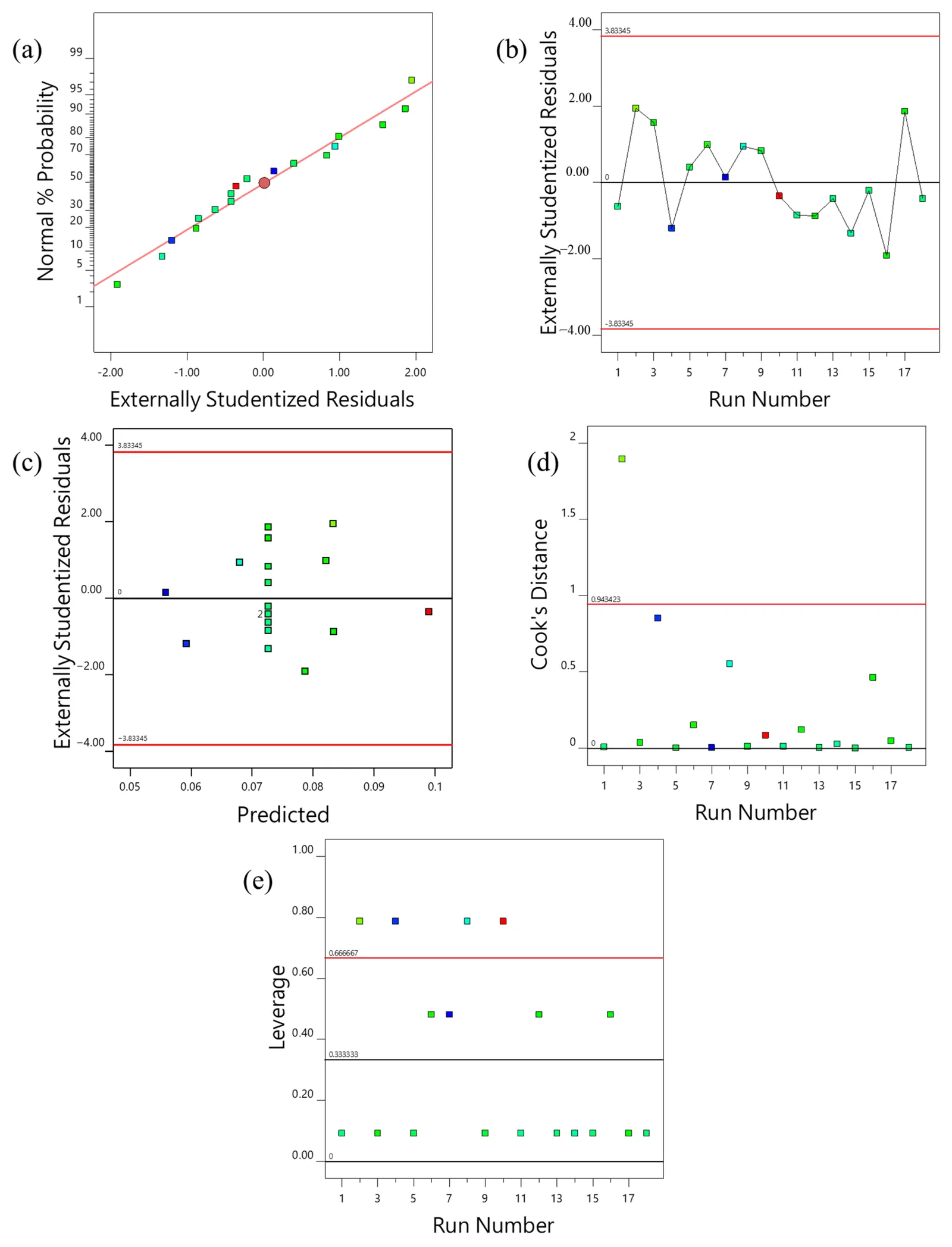
Residual diagnostics of the model: (**a**) Normal probability plot of residuals; (**b**) Residuals vs. run number; (**c**) Residuals vs. predicted; (**d**) Cook’s distance plot; (**e**) Leverage plot.

The residual diagnostics of the model are shown in Figure 7. Figure 7a presents the normal probability plot of residuals, where the points are approximately distributed along a straight line, indicating that the residuals follow a normal distribution. Figure 7b shows the residuals vs. run number plot, where each data point is labeled with its corresponding run number; the studentized residuals are randomly distributed within ±2 and do not approach the ±3.83345 control limits, indicating that the residuals satisfy the independence assumption. Figure 7c presents the residuals vs. predicted plot, where the residuals are randomly scattered around the zero line, and all studentized residuals are within ±2, with no discernible trend or heteroscedasticity, indicating that the residuals satisfy the homoscedasticity assumption. Figure 7d shows the Cook’s distance plot; only Run 2 has a Cook’s distance of approximately 1.8, slightly above the conventional threshold of 1, but its studentized residual falls within an acceptable range, and the remaining points have Cook’s distances below 1. Figure 7e presents the leverage plot; four experimental points have leverage values of approximately 0.8, slightly above the conventional warning threshold of 0.6667. These points correspond to the corner points of the CCF design space (extreme values of layer height and line width), where inherently high leverage is a characteristic feature due to their edge positions in the design space, and their residuals are all within acceptable ranges, thus not materially affecting the model conclusions. Collectively, these diagnostic results confirm that the model residuals satisfy the assumptions of normality, independence, and homoscedasticity, with no influential points that would materially affect the model conclusions, demonstrating the robustness of the established quadratic regression model.

The ANOVA results for the quadratic model are presented in Table 6.

As shown in Table 6, the *p*-value of the final model was less than 0.0001, indicating that the model was statistically highly significant. Meanwhile, the lack-of-fit *p*-value was greater than 0.05 (non-significant), indicating that the model fit was adequate and no systematic bias was observed, confirming the model’s usability. Regarding the significance of individual factors, A, AB, A^2^, and B^2^ all showed highly significant effects on straightness error (*p* < 0.01), while factor B exhibited a significant effect (0.05 < *p* < 0.1). The coefficient of determination R^2^ of the model was 0.9787, and the adjusted R^2^ was as high as 0.9698, indicating that the model can explain over 96% of the response variation, demonstrating excellent fitting accuracy and predictive reliability. The coefficient of variation (CV) of the model was 2.21%, far below the engineering acceptance threshold of 10%, indicating high prediction accuracy and good experimental repeatability. The adequate precision of the model was 45.4527, far greater than the critical value of 4 recommended by Design-Expert, indicating that the regression model possesses exceptionally strong signal identification capability and can be used to predict response variations within the design space.

**Figure 8:**
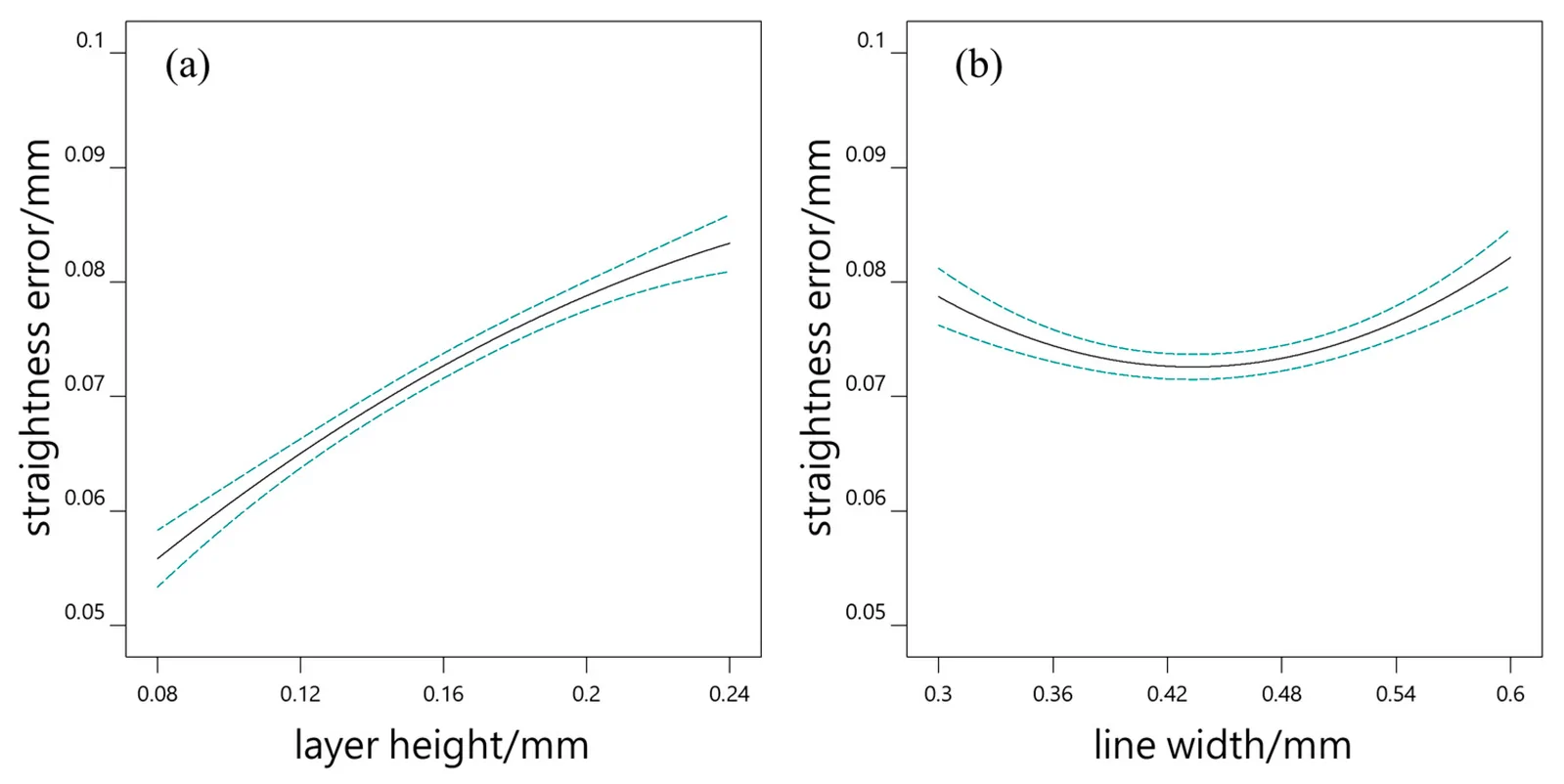
Main effects of factors A and B on straightness error: (**a**) Main effect of factor A (layer height). (**b**) Main effect of factor B (line width).

The main effects of the significant factors on straightness error are shown in Figure 8. Figure 8a indicates that straightness error decreased with decreasing levels of factor A (layer height). This can be attributed to two aspects: first, a thinner layer height effectively reduces the “staircase effect” on vertical sidewalls, resulting in a smoother surface and better straightness at the macroscopic level; second, the smaller volume of plastic deposited in thin layers cools more rapidly, and when subsequent molten material is extruded onto its surface, slight localized remelting occurs, which promotes interlayer fusion, blurs the boundary lines, and thus improves surface profile continuity. Figure 8b shows that straightness error exhibited a non-linear (initially decreasing and then increasing) trend with line width variation. This is essentially the result of the superposition of two different types of defects during the FDM process. When the line width is narrow, the extruded filament is extremely thin, and factors such as nozzle pressure fluctuations, tensile instability of the molten PLA, and localized stresses from cooling shrinkage cause significant random disturbances to the filament edges, resulting in uneven edges and high straightness error. As line width increases, the filament becomes thicker, and these small random defects are effectively averaged out and suppressed; the extrusion flow stabilizes, edges become smoother, and error decreases. However, when line width increases further, systematic deformation becomes dominant: wider lines entail greater extrusion volume and higher heat accumulation, leading to significantly enhanced internal stresses and non-uniform shrinkage of the PLA material during layer-by-layer stacking and cooling. At the same time, wider lines are more sensitive to long-wavelength distortions such as uneven print bed, micro-vibrations of the *Z*-axis, and low-speed jitter in the nozzle path. These global bending and warpage effects cannot be smoothed by increasing line width; instead, they are amplified, causing straightness error to rise again.

**Figure 9:**
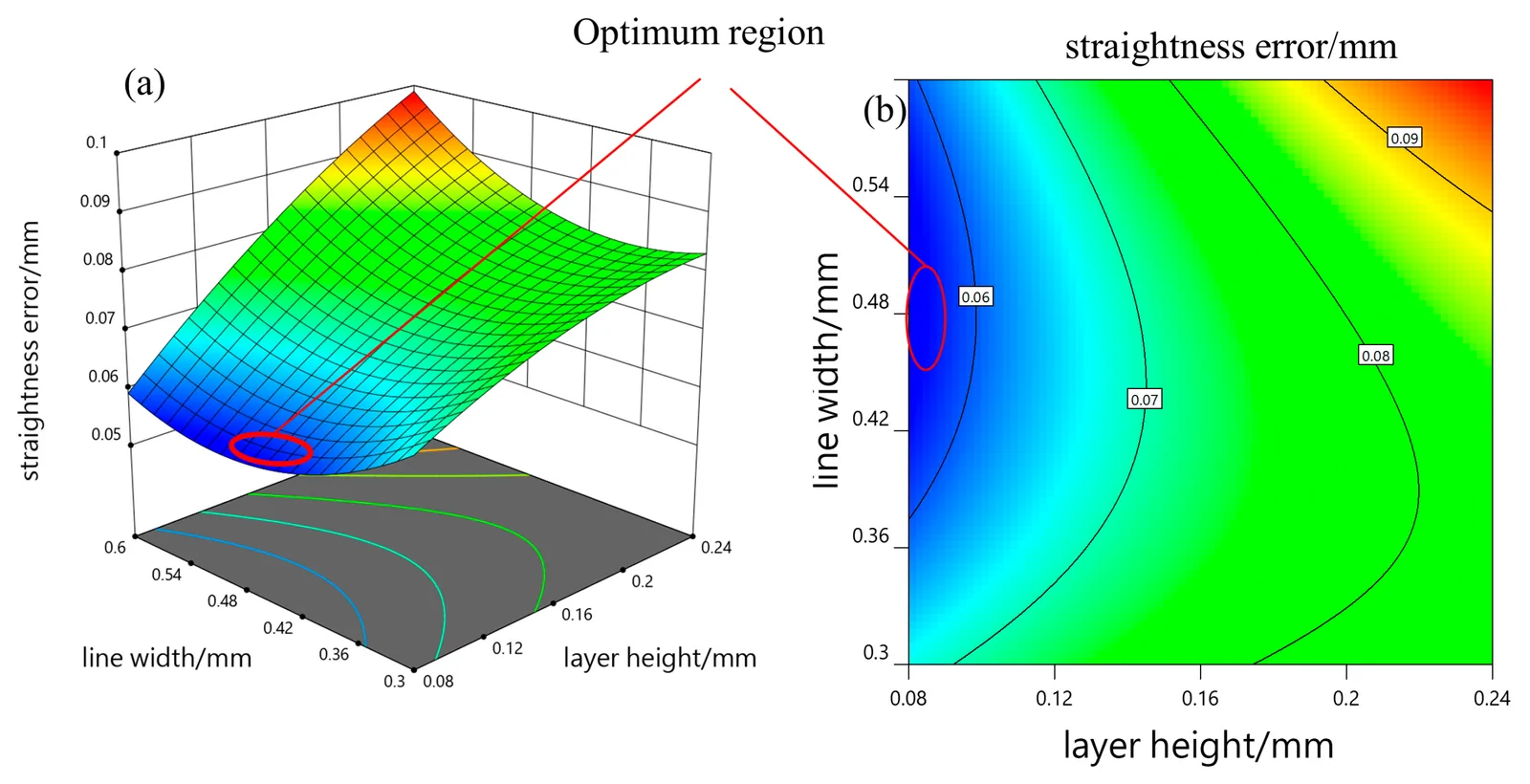
Response surface plot of the A–B interaction effect: (**a**) 3D response surface. (**b**) Contour plot.

The significant interaction effect between line width and layer height on straightness error is shown in Figure 9. This effect can be attributed to the filament spreading morphology and thermal shrinkage behavior jointly determined by the two factors. During FDM, the relative relationship between line width and nozzle diameter (0.4 mm) determines the lateral spreading of the molten filament on the deposition surface, while layer height constrains the vertical compression ratio. Together, they define the cross-sectional geometry of the filament, which in turn determines the edge quality. Line width and layer height do not independently affect straightness; rather, they act synergistically through the coupling variable “spreading ratio” (line width-to-layer height ratio). When the spreading ratio is too small, vertical compression is insufficient, the cross-section tends toward circular, lateral flow is restricted, and edges are susceptible to irregular undulations caused by extrusion pressure fluctuations. When the spreading ratio is too large, the filament is excessively flattened, heat accumulation increases, and cooling shrinkage becomes non-uniform, exacerbating macroscopic edge curvature. Only at a moderate spreading ratio can the filament achieve sufficient lateral spreading to form a regular flat cross-section while avoiding shrinkage instability caused by excessive heat input, thus achieving a balance between rheological spreading and thermal contraction. Furthermore, a moderate line width (approximately 0.45–0.50 mm, i.e., 1.1–1.25 times the nozzle diameter) generally falls within this equilibrium range under typical process conditions, although the specific optimum may shift within a certain range depending on actual printing conditions such as material viscosity, ambient heat dissipation, and platform adhesion state. Consequently, the minimization of straightness error occurs in the synergistic region of small layer height and moderate spreading ratio, where filament spreading is uniform, cooling is rapid, and internal stress distribution is most favorable.

### 3.3. Prediction Model Construction and Comparison

Under the condition of small-sample experimental data (the training set in this study consisted of only 18 groups), the selection of modeling methods requires a trade-off between fitting capability and overfitting risk. Among common machine learning methods, artificial neural networks (ANN) possess strong nonlinear fitting capability, but their successful application typically relies on thousands of training samples. When the sample size is insufficient, ANN is highly prone to overfitting; that is, the model performs excellently on the training set, but its generalization capability deteriorates sharply on independent test sets [31,32].

In contrast, decision trees have lower sample size requirements, and their tree-like structure offers good interpretability, intuitively presenting the hierarchical decision logic among factors. More importantly, by introducing a genetic algorithm (GA) for global optimization of decision tree hyperparameters (minimum leaf size and maximum number of splits), overfitting risk can be effectively mitigated under small-sample conditions while maintaining high prediction accuracy [33]. Therefore, this study adopts DT + GA as a representative data-driven model and compares it with the CCF-prior-based RSM model to investigate the applicability and advantages of parametric versus non-parametric models in small-sample CCF experimental scenarios.

The 18 sets of CCF experimental data were used as the training set to establish both the RSM regression equation and the DT + GA prediction model. For DT + GA, leave-one-out cross-validation (LOO-CV) was employed to internally partition the validation set within the training data for genetic algorithm hyperparameter optimization. RSM, which has no tunable hyperparameters, did not require cross-validation. Finally, five independent reserved experimental data points were used as the test set to evaluate and compare the predictive generalization capabilities of the two models. Both models were compared on the same external test set in terms of MSE and MAE to ensure fairness and transparency of the evaluation.

#### 3.3.1. Response Surface Prediction Model

Based on the CCF experimental data, a non-linear regression prediction model for straightness error (y) as a function of the key process parameters (A and B) was established through fitting in Design-Expert software, as shown in Equation (1).

y = 0.133994 + 0.097801·A − 0.38·B + 0.506944·A·B − 0.480324·A^2^ + 0.344856·B^2^ (1)

In Equation (1), A is the layer height (mm), and B is the line width (mm), both using actual values.

The five test experiments were used to validate the predictive capability of the model. MAE was employed to evaluate the average prediction accuracy because it shares the same dimension as the response variable, facilitating engineering interpretation. MSE was simultaneously used to assess model stability and sensitivity to outliers, ensuring that no individual severely deviating predictions existed during the prediction process. The results are shown in Table 7.

#### 3.3.2. DT + GA Prediction Model

The input variables of the DT + GA model were layer height (A) and line width (B), and the output variable was straightness error. The decision tree adopted the CART (Classification and Regression Trees) algorithm with MSE as the node-splitting criterion. The genetic algorithm was used to optimize two key hyperparameters of the decision tree: minimum leaf size (MinLeafSize) and maximum tree depth (MaxDepth). Through the joint optimization of the genetic algorithm and decision tree, model complexity and generalization capability were effectively balanced to avoid small-sample overfitting caused by excessively deep tree structures.

The genetic algorithm parameters were set as follows: population size = 50, maximum generations = 100, and crossover fraction = 0.8. Since the optimization variables involved integer constraints (both MinLeafSize and MaxDepth are positive integers), the genetic algorithm automatically adopted mutation operators suitable for integer programming. The fitness function was defined as the leave-one-out cross-validation mean squared error (LOO-CV MSE), which the genetic algorithm aimed to minimize. The optimization search ranges were MinLeafSize (1, 10)and MaxDepth (3, 20).

During the 100-generation iteration process, the leave-one-out cross-validation (LOO-CV) mean squared error (MSE) stabilized at the early stage of iteration, indicating that the genetic algorithm exhibits high search efficiency and is capable of rapidly identifying a stable set of hyperparameters within the given search space. The optimal hyperparameters ultimately determined by the algorithm were a minimum leaf size (MinLeafSize) of 1 and a maximum tree depth (MaxDepth) of 9, with a corresponding LOO-CV MSE of 3.946 × 10^−5^. This result demonstrates that the genetic algorithm can independently and effectively accomplish the automatic optimization of decision tree hyperparameters within the specified search space, without relying on empirical manual tuning or trial-and-error. This ensures the objectivity and reproducibility of the model structure selection, and provides algorithmic support for safeguarding the generalization performance of decision tree models under small-sample conditions.

#### 3.3.3. Comparison of the Two Prediction Models

Table 8 compares the prediction accuracy of RSM and DT + GA on both the training set and the test set. On the 18-group training set, RSM achieved a coefficient of determination R^2^ of 0.9787, while DT + GA achieved an R^2^ of 0.8228, indicating that RSM exhibited significantly superior goodness-of-fit to the training data compared to DT + GA. The MSE and MAE of RSM were 1.80 × 10^−6^ mm^2^ and 0.001150 mm, respectively, both outperforming those of DT + GA (1.497 × 10^−5^ mm^2^ and 0.002709 mm), demonstrating that the CCF-prior-based quadratic response surface model achieved higher approximation accuracy during the fitting stage, with its MSE accounting for approximately 12.0% of that of DT + GA.

**Figure 10:**
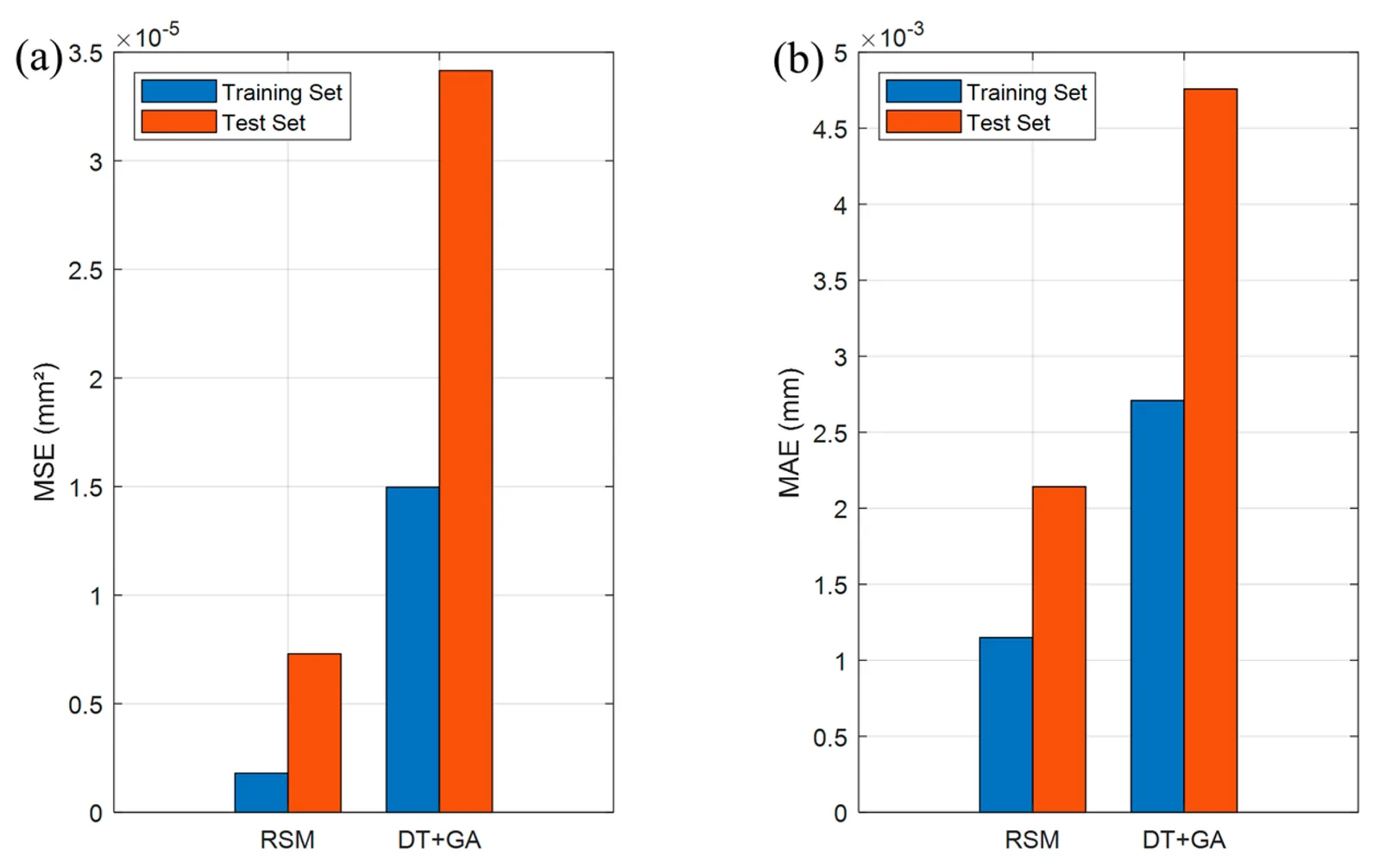
MSE and MAE of the two prediction models: (**a**) MSE. (**b**) MAE.

On the five independent test sets, RSM achieved an MSE of 7.29 × 10^−6^ mm^2^ and an MAE of 0.002142 mm, both lower than those of DT + GA (MSE = 3.415 × 10^−5^ mm^2^, MAE = 0.004578 mm). As can be clearly observed from Figure 10, RSM outperformed DT + GA in terms of both MSE and MAE on the test set, with relative reductions of approximately 78.7% in MSE and 53.2% in MAE. These results further confirm that, in small-sample CCF experimental scenarios, the parametric model based on a statistical prior (RSM) achieves higher prediction accuracy and stronger generalization capability than the data-driven non-parametric model (DT + GA).

The advantage of RSM stems from the fact that the CCF experimental design inherently matches the quadratic surface assumption, enabling the precise characterization of the continuous mapping relationship between layer height (A), line width (B), and straightness error through least-squares fitting with only six coefficients to be estimated. In contrast, the decision tree model adopts a recursive partitioning strategy, approximating the smooth response surface with stepwise constants, which inherently introduces considerable discretization errors even during the training stage, thereby limiting its generalization capability.

## 4. Optimization of Process Parameters and Experimental Validation

### 4.1. Optimization of Process Parameters

Based on the established quadratic regression model (Equation (1)), with the objective of minimizing the predicted straightness error, constrained convex optimization was performed using the MATLAB R2020b fmincon function (MathWorks, Inc., Natick, MA, USA) within the ranges of layer height (0.08, 0.24) mm and line width (0.30, 0.60)mm. The optimal process parameter combination was determined as: layer height = 0.08 mm, line width = 0.49 mm, at which the model predicted a minimum straightness error of y_min_ = 0.0552 mm.

### 4.2. Experimental Validation

**Figure 11:**
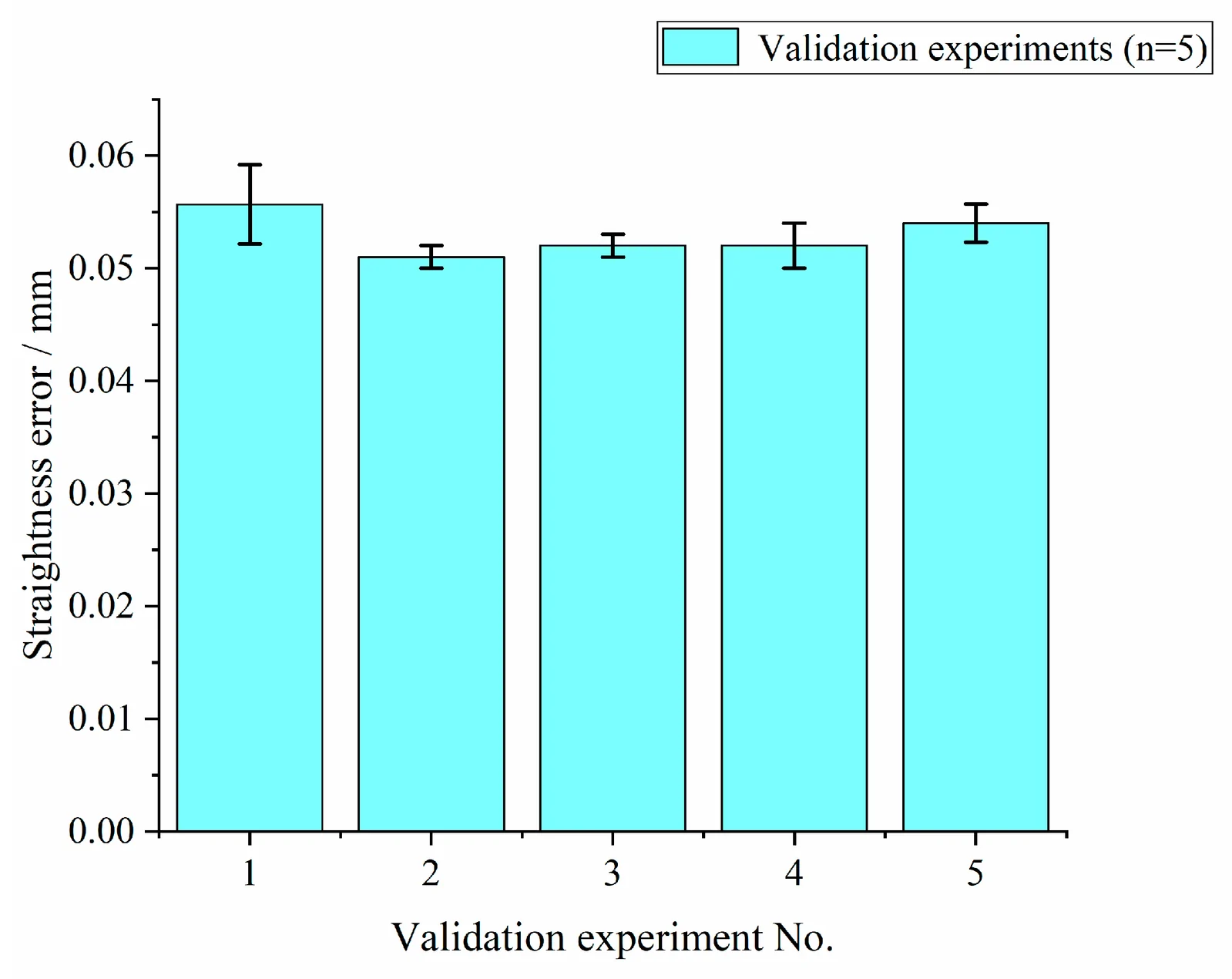
Validation results of straightness error under optimal process parameters.

To verify the reliability of the optimization results, five repeated specimens were fabricated under the optimal process parameters (layer height = 0.08 mm, line width = 0.49 mm), and their straightness errors along the height direction were measured, as shown in Figure 11. The measured straightness errors of the five validation specimens were 0.055667, 0.051, 0.052, 0.052, and 0.054 mm, respectively, with an overall mean of 0.0529 mm, a standard deviation of 0.0018 mm, and a coefficient of variation (CV) of 3.5%, indicating good repeatability and process stability of the optimal parameters. The mean validation result (0.0529 mm) was slightly lower than the model-predicted value (0.0552 mm), with a relative deviation of 4.2%, demonstrating good agreement between the predicted and experimental values. The fact that the predicted value is slightly higher than the measured mean confirms that the regression model provides reliable prediction accuracy near the optimum, and can serve as a practical guideline for process optimization of straightness error in FDM-printed parts.

## 5. Discussion

### 5.1. Efficiency and Risk Control of the Two-Stage Strategy

This study adopted a “PB screening + CCF modeling” stepwise strategy, reducing the parameter dimension from nine to two factors and requiring only 18 experimental runs for subsequent modeling, thereby significantly lowering trial-and-error costs. However, the PB design is a resolution-limited screening design in which main effects may be confounded with interaction effects. To mitigate this risk, this study re-validated the significance of A, B, and their interaction AB in the CCF stage (all *p* < 0.01), confirming that the screening results were robust and reliable under the current experimental conditions. It should also be noted that the seven screened-out parameters showed insignificant main effects within their set ranges, but this does not exclude the possibility that they may indirectly affect straightness error through interactions with other parameters or over a wider range of parameter settings. Future work may therefore design dedicated factorial experiments targeting these parameters to more comprehensively reveal their potential influence mechanisms.

### 5.2. Mechanism Analysis

The results presented in Section 3.2 indicate that layer height is the dominant factor affecting straightness, while line width exhibits a non-linear effect, and their interaction is statistically significant. This behavior can be interpreted from thermodynamic and rheological perspectives.

Layer height determines the deposited volume and cooling rate of each layer. A smaller layer height allows the filament to cool more rapidly below the glass transition temperature after deposition, limiting the relaxation time of polymer molecular chains and thereby reducing residual stress accumulation caused by thermal shrinkage [34]. In addition, the reduced temperature gradient between adjacent layers in thin-layer printing helps minimize warpage deformation and improves edge straightness in the Z-direction [35].

The non-linear effect of line width on straightness arises from the competition between two distinct mechanisms. When the line width is too narrow, the extruded filament is subjected to a large draw ratio, leading to flow front instability and edge fluctuation [36]. When the line width is too wide, the deposition volume per unit length increases, enhancing heat accumulation, and non-uniform cooling-induced shrinkage stress becomes dominant, causing macroscopic edge curvature [37]. Therefore, an optimal range of line width exists where extrusion flow remains stable and thermal shrinkage is uniform.

The interaction between layer height and line width can be unified through the spreading ratio (line width-to-layer height ratio), as detailed in Section 3.2. Layer height plays the most significant role in line aspect ratios and void formation [38]. An appropriate spreading ratio allows sufficient lateral spreading to form a regular flat cross-section [39] while avoiding shrinkage instability caused by excessive heat input [37].

### 5.3. Recommended Process Parameters

The model is applicable to PLA Matte material with layer heights ranging from 0.08 to 0.24 mm and line widths from 0.30 to 0.60 mm. The effect of layer height on straightness (F = 421.84) far exceeds that of line width (F = 6.59), indicating that layer height is the dominant factor in controlling straightness error. The global optimum obtained by differentiation of the regression equation is A = 0.08 mm with B ≈ 0.49 mm. Therefore, for slender parts with stringent straightness requirements, it is recommended to prioritize controlling layer height to no more than 0.12 mm, with line width set near 0.49 mm.

From an engineering perspective, the practical significance of these recommendations lies in their direct applicability to the production of FDM-printed parts with stringent straightness requirements, such as guide rails, sliders, and other kinematic components. The quantified relationship between process parameters and straightness error allows engineers to pre-select appropriate parameters based on specific accuracy requirements without extensive trial-and-error. The dominant effect of layer height also provides a clear priority guideline: for applications demanding high motion accuracy, layer height should be reduced as much as possible within the feasible range, while line width can be adjusted for secondary fine-tuning.

### 5.4. Comparison with Existing Studies

To further clarify the relative level of the process optimization results and prediction model accuracy achieved in this study compared to similar studies, the key conclusions are systematically compared with the existing literature.

#### 5.4.1. Consistency of Optimal Process Parameters

The key parameters screened in this study (layer height and line width) and their optimal ranges (layer height ≈ 0.08 mm, line width ≈ 0.49 mm) are in reasonable agreement with existing studies, but also exhibit differences arising from specific evaluation metrics. Jayakumar et al. [10] identified layer height as the decisive parameter affecting the surface roughness of PLA parts, with the optimal layer height also at a relatively low level (≈ 0.10 mm), consistent with the findings of this study, suggesting that thin-layer deposition has universal applicability for simultaneous improvement of surface quality and shape accuracy. Khraisat et al. [11] further found significant interaction effects among layer height, infill pattern, and printing temperature, but that study used surface roughness (Ra) as the sole response variable and did not address shape tolerances. The present study complements these findings by quantifying the main effect of layer height on straightness error (F = 421.84), quantitatively verifying the dominant role of layer height in shape accuracy and extending the cognitive boundaries of previous studies into the dimension of shape tolerances. Regarding the effect of line width, Arreda et al. [12] reported a non-linear effect of line width on the dimensional accuracy of PLA parts, but did not further analyze the causes of this non-linearity. Through high-resolution CCF experimental data, the present study reveals the “U-shaped” non-linear mechanism by which line width affects straightness error: random disturbances dominate at narrow line widths (extrusion fluctuations, tensile instability), while systematic deformation dominates at wide line widths (heat accumulation, shrinkage instability). This mechanistic analysis provides a theoretical basis for precise setting of the process window.

#### 5.4.2. Applicability of Small-Sample Modeling Strategies

RSM achieved superior prediction accuracy to DT + GA under small-sample (18 groups) conditions. Decision tree models are generally considered to have strong capability for capturing non-linear relationships. However, when the sample size is limited, data-driven models are prone to overfitting, and their generalization performance may deteriorate [31,32]. In contrast, parametric models such as RSM leverage prior knowledge of the response surface structure. The results of this study show that when experimental design prior information is sufficient (i.e., the CCF design inherently matches the quadratic surface assumption), the least-squares estimation of parametric models is more efficient than the piecewise constant approximation of non-parametric models. This finding suggests that in small-sample experimental scenarios, the quality of experimental design may be more critical than algorithm selection; a high-quality CCF design itself provides sufficient geometric information about the response surface, and the prediction efficiency of RSM, which shares the same assumptions as the design, tends to outperform that of general-purpose data-driven machine learning models.

### 5.5. Outlook

(1) This study was conducted using only PLA material. Future work could extend the method to other thermoplastics such as ABS and PETG to investigate the influence of material properties on model applicability. (2) Straightness error is only one of several geometric tolerances. Future research could incorporate multi-dimensional geometric tolerance indicators such as cylindricity and parallelism to establish multi-objective optimization models for coordinated control of the comprehensive accuracy of FDM-printed parts. (3) Although the CCF experimental design has effectively supported the modeling requirements of this study, the sample size remains relatively small. Future work could combine active learning or transfer learning methods to further improve model prediction accuracy under limited experimental samples.

## 6. Conclusions

This study systematically investigated the effects of FDM process parameters on the straightness error of PLA printed parts along the height direction through PB-CCF experimental design and prediction model comparison. The main quantitative findings are as follows: the optimal process parameters (layer height = 0.08 mm, line width = 0.49 mm) yield a minimum predicted straightness error of 0.0552 mm; the established quadratic regression model achieves a coefficient of determination R^2^ = 0.9787, a coefficient of variation CV = 2.21%, and a signal-to-noise ratio of 45.45; and the validation experiments show a relative deviation of 4.2% between the predicted and measured values.

(1) Through Plackett–Burman screening experiments, two key factors significantly affecting straightness error—layer height (A) and line width (B)—were identified from nine candidate process parameters, with layer height showing the most significant effect (*p* < 0.01). The remaining seven parameters had no significant effect on straightness error within the experimental ranges and can be fixed in subsequent optimization, thereby effectively simplifying the process control dimensions.
(2) ANOVA of the quadratic response surface model established based on the face-centered central composite design (CCF) showed that layer height, line width, and their interaction and quadratic terms all had significant effects on straightness error. Smaller layer height resulted in smaller straightness error, and line width near the center level yielded the lowest straightness error. These results confirm the high fitting accuracy and effectiveness of the model for straightness error prediction and process parameter optimization.
(3) Comparison of RSM and DT + GA prediction models on 18 sets of CCF training data and 5 independent test sets showed that RSM achieved test set MSE and MAE of 7.29 × 10^−6^ mm^2^ and 0.002142 mm, respectively, both superior to those of DT + GA (MSE = 3.415 × 10^−5^ mm^2^, MAE = 0.004578 mm). Meanwhile, RSM achieved a training set R^2^ of 0.9787, significantly higher than DT + GA’s 0.8228. RSM maintained better prediction performance on both training and test sets, demonstrating that the response surface method based on a CCF design prior has higher modeling efficiency and generalization reliability for small-sample FDM straightness error prediction.
(4) In practical FDM printing, it is recommended to prioritize control of layer height near 0.08 mm and line width near 0.49 mm to achieve optimal straightness accuracy.

## Figures and Tables

**Table 1:** Factor levels for PLA screening experiments.

Level	A/mm	B/mm	C	D/%	E/°	F/(mm/s)	G/-	H/°C	J/°C
−1	0.08	0.3	2	10	15	60	0.98	195	55
+1	0.24	0.6	6	50	45	200	1.02	225	65

Note: The parameter level ranges were determined based on the equipment manufacturer’s recommendations and previous studies [10,12,18].

**Table 2:** Factor levels for straightness CCF experiments.

Level	A/mm	B/mm
−1	0.08	0.30
0	0.16	0.45
+1	0.24	0.60

**Table 3:** ANOVA for straightness error in the PB experiment.

Source	Sum of Squares	df	Mean Squared	F-Value	*p*-Value	Significance
Model	0.0052	2	0.0025	23.07	0.0003	Significant
A	0.0044	1	0.0042	39.41	0.0001	
B	0.0008	1	0.0007	6.73	0.0290	
Residual	0.0010	9	0.0001			
Total	0.0061	11				
R^2^ = 0.8368 R^2^adj = 0.8005 Adeq Precision = 10.2452						

**Table 4:** CCF experimental scheme for straightness.

Std	Run	A (Layer Height)	B (Line Width)	Straightness Error/mm
1	8	−1	−1	0.069
2	2	1	−1	0.085
3	4	−1	1	0.058
4	10	1	1	0.099
5	7	−1	0	0.056
6	12	1	0	0.082
7	16	0	−1	0.077
8	6	0	1	0.083
9	14	0	0	0.071
10	9	0	0	0.074
11	1	0	0	0.072
12	15	0	0	0.072
13	17	0	0	0.075
14	13	0	0	0.072
15	11	0	0	0.071
16	3	0	0	0.075
17	18	0	0	0.072
18	5	0	0	0.073

Note: The coded levels −1, 0, +1 for factors A and B correspond to the actual values listed in Table 2.

**Table 5:** Comparison of performance parameters for each model.

Source	Sequential *p*-Value	Lack of Fit *p*-Value	Adjusted R^2^	Predicted R^2^
Linear	<0.0001	<0.0001	0.7289	0.4882
2FI	0.0078	0.0003	0.8278	0.5925
Quadratic	<0.0001	0.3248	0.9698	0.9198
Cubic	0.2004	0.5352	0.9737	0.9083

**Table 6:** ANOVA for straightness error in the CCF experiment.

Source	Sum of Squares	df	Mean Squared	F-Value	*p*-Value	Significance
Model	0.0015	5	0.0003	110.22	<0.0001	significant
A-A	0.0011	1	0.0011	421.84	<0.0001	
B-B	0.0000	1	0.0000	6.59	0.0247	
AB	0.0001	1	0.0001	54.82	<0.0001	
A^2^	0.0000	1	0.0000	10.50	0.0071	
B^2^	0.0002	1	0.0002	66.90	<0.0001	
Residual	0.0000	12	2.700 × 10^−6^			
Lack of Fit	9.945 × 10^−6^	3	3.315 × 10^−6^	1.33	0.3248	not significant
Pure Error	0.0000	9	2.495 × 10^−6^			
Cor Total	0.0015	17				

**Table 7:** Prediction errors of the RSM model on the test set.

Test Point	Experimental Value/mm	Predicted Value/mm	Absolute Error/mm	Squared Error/mm^2^
1	0.073667	0.069313	0.004354	0.00001896
2	0.080667	0.076890	0.003777	0.00001426
3	0.065333	0.066324	0.000991	0.000000982
4	0.081333	0.081250	0.000083	0.0000000069
5	0.060667	0.062147	0.001480	0.000002190
MSE/mm^2^	0.00000728			
MAE/mm	0.002137			

**Table 8:** Comparison of prediction accuracy of different models.

Model	R^2^ (Training Set)	MSE (Training Set)/mm^2^	MAE (Training Set)/mm	MSE (Test Set)/mm^2^	MAE (Test Set)/mm
RSM	0.9787	0.00000180	0.001150	0.00000729	0.002142
DT + GA	0.8228	0.00001497	0.002709	0.00003415	0.004578

## Data Availability

The original contributions presented in this study are included in the article. Further inquiries can be directed to the corresponding authors.

## Abbreviations

The following abbreviations are used in this manuscript:

FDM	Fused Deposition Modeling
PLA	Polylactic Acid
PB	Plackett–Burman
CCD	Central Composite Design
CCF	Face-Centered Central Composite Design
RSM	Response Surface Methodology
DT	Decision Tree
GA	Genetic Algorithm
DT + GA	Decision Tree–Genetic Algorithm
ANOVA	Analysis of Variance
MSE	Mean Squared Error
MAE	Mean Absolute Error
LOO-CV	Leave-One-Out Cross-Validation
CART	Classification and Regression Trees
CV	Coefficient of Variation
